# Improving Stability and Specificity of CRISPR/Cas9 System by Selective Modification of Guide RNAs with 2′-fluoro and Locked Nucleic Acid Nucleotides

**DOI:** 10.3390/ijms232113460

**Published:** 2022-11-03

**Authors:** Lubov Sakovina, Ivan Vokhtantsev, Mariya Vorobyeva, Pavel Vorobyev, Darya Novopashina

**Affiliations:** 1 Institute of Chemical Biology and Fundamental Medicine SB RAS, 630090 Novosibirsk, Russia; 2 Faculty of Natural Sciences, Novosibirsk State University, 630090 Novosibirsk, Russia

**Keywords:** CRISPR, crRNA, tracrRNA, sgRNA, gene editing, 2’-modification, LNA, 2’-fluoro RNA, 2’-O-methyl RNA, off-target effect

## Abstract

The genome editing approach using the components of the CRISPR/Cas system has found wide application in molecular biology, fundamental medicine and genetic engineering. A promising method is to increase the efficacy and specificity of CRISPR/Cas-based genome editing systems by modifying their components. Here, we designed and chemically synthesized guide RNAs (crRNA, tracrRNA and sgRNA) containing modified nucleotides (2’-O-methyl, 2’-fluoro, LNA—locked nucleic acid) or deoxyribonucleotides in certain positions. We compared their resistance to nuclease digestion and examined the DNA cleavage efficacy of the CRISPR/Cas9 system guided by these modified guide RNAs. The replacement of ribonucleotides with 2’-fluoro modified or LNA nucleotides increased the lifetime of the crRNAs, while other types of modification did not change their nuclease resistance. Modification of crRNA or tracrRNA preserved the efficacy of the CRISPR/Cas9 system. Otherwise, the CRISPR/Cas9 systems with modified sgRNA showed a remarkable loss of DNA cleavage efficacy. The kinetic constant of DNA cleavage was higher for the system with 2’-fluoro modified crRNA. The 2’-modification of crRNA also decreased the off-target effect upon in vitro dsDNA cleavage.

## 1. Introduction

Adaptive immune defense systems of bacteria use clustered regularly interspaced short palindromic repeats/CRISPR-associated protein (CRISPR/Cas) systems to resist foreign invading nucleic acids [1,2]. The CRISPR/Cas9 technology has become a potent instrument for altering the genomes and found extensive applications in molecular biology and genetic engineering [3,4,5,6,7,8,9].

This approach relies on site-directed cleavage of dsDNA by the CRISPR/Cas9 system, with further repair of the resulting double-stranded break via nonhomologous end joining (NHEJ) or homology-directed repair (HDR) mechanism that lead to local structure changes in the gene: insertions or deletions (indels) or nucleotide substitutions. The process is realized by an effector complex, including the Cas9 protein with RNA-dependent DNA-endonuclease activity, the mature guide crispr RNA (crRNA) and trans-activating crispr RNA (tracrRNA). The complex recognizes a double-stranded target DNA containing a protospacer flanked by protospacer adjacent motif 5′-NGG-3′ (PAM) at the 3′-end. Then, the cleavage of the target DNA occurs at a distance of three nucleotides from the PAM.

J. Doudna and co-authors [10] proposed to combine a pair of crRNA and tracrRNA into the chimeric single guide RNA. Undoubtedly, this is a winning strategy for the delivery of guide RNA by the DNA vector, but it seems to be less advantageous in the case of chemically synthesized guide RNAs. Furthermore, a pair of guide RNAs (crRNA + tracrRNA) provides better efficacy and a higher rate of target DNA cleavage than sgRNA [10].

Increasing the efficacy and specificity of CRISPR/Cas-based genome editing systems for practical applications remains an acute task. A prospective way to solve this problem is to modify the components of CRISPR/Cas systems, particularly guide RNAs [6,7,8,9,11,12,13]. Chemically modified guide RNAs have an increased nuclease resistance compared to their unmodified natural analogs. Moreover, selective chemical modifications of guide RNAs permit increasing the efficacy and duration of action of the CRISPR/Cas9 system in cells. The various variants of ribonucleotide modifications in guide RNA structure include 2’-fluorinated nucleotides [14,15], 2’-O-methylated ribonucleotides [14], LNA nucleotides (LNA—locked nucleic acid) [16] and replacement of ribonucleotides with deoxyribonucleotides [9,10,17,18,19].

Usually, the efficacy of CRISPR/Cas9 system action is examined on cell culture containing incorporated green fluorescent protein (GFP) or enhanced green fluorescent protein (EGFP) gene and estimated by the change of fluorescence of the cognate protein in the cells [14,18]. This approach is resource and time consuming and gives no information on the rate constant of DNA cleavage. With this in mind, we chose the cell-free model system in our work.

In this study, we designed guide RNAs (crRNA, tracrRNA and sgRNA) containing modified nucleotides (2’-O-methyl, 2’-fluoro, LNA—locked nucleic acid) or deoxyribonucleotides in certain positions to increase their resistance to nuclease digestion (Table S1). We investigated in vitro the nuclease stability and functional activity of the modified guide RNAs and studied the influence of 2’-modifications on the kinetic parameters of DNA cleavage by the CRISPR/Cas9 system and on possible off-target effects. 

## 2. Results and Discussion

### 2.1. Design and Synthesis of Guide RNAs and Their 3’-Fluorescein Conjugates

**Figure 1:**
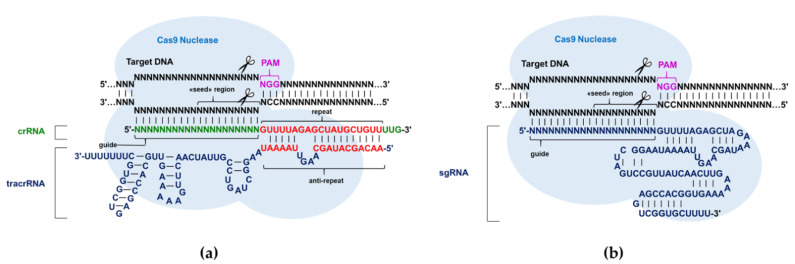
The *Streptococcus pyogenes* Cas9 enzyme guided by RNA cleaving each strand of a DNA target site next to the protospacer adjacent motif (PAM): (**a**) dual-RNA-guided (crRNA and tracrRNA) CRISPR/Cas9 system; (**b**) sgRNA-guided CRISPR/Cas9 system.

**Figure 2:**
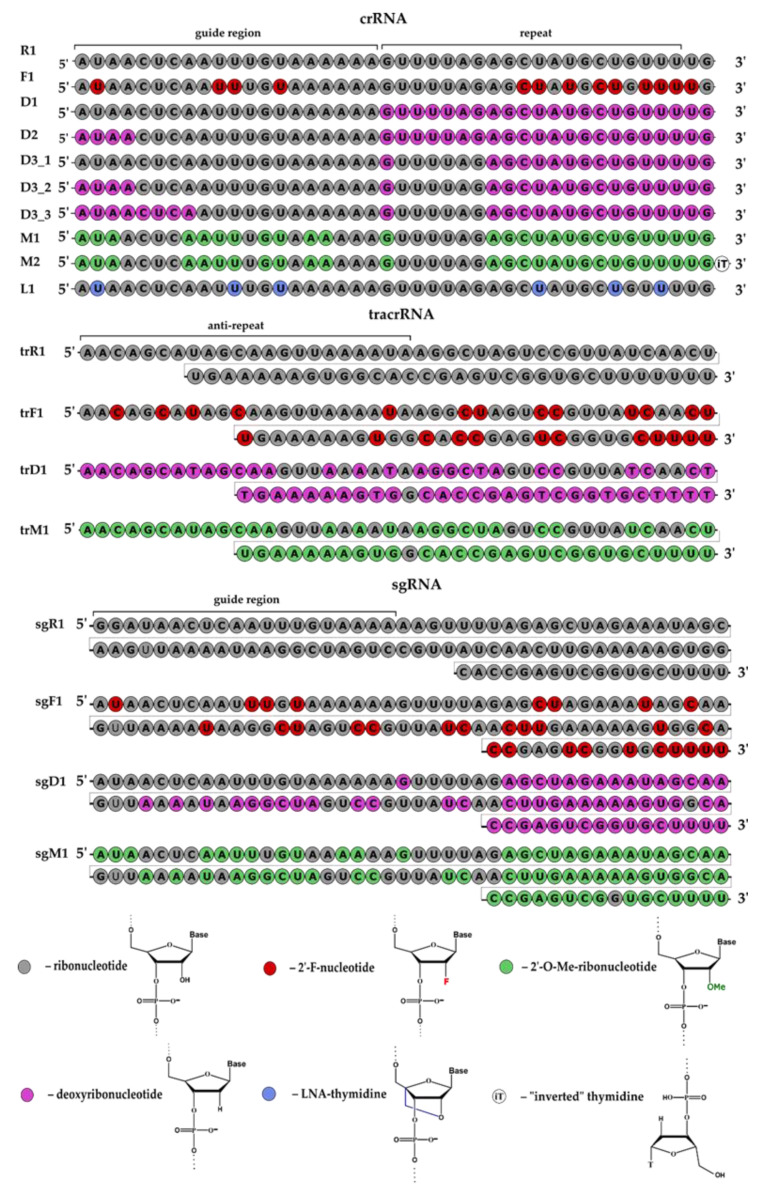
The structures of modified guide RNAs used as components of CRISPR/Cas9 system for DNA cleavage in this investigation. All ribonucleotide substitutions are shown with colored circles (see the legend above).

In this study, we investigated the influence of ribonucleotide replacement in guide RNAs with modified ribo- or deoxyribonucleotides on nuclease resistance and efficacy of CRISPR/Cas9 action using an in vitro system. We employed the *Streptococcus pyogenes* Cas9 nuclease in two variants of in vitro systems containing a pair of guide RNAs (crRNA and tracrRNA) or single chimeric guide RNA (sgRNA) (Figure 1). In the first stage, we designed and synthesized guide RNAs (crRNA, tracrRNA and sgRNA), their modified analogs and 3’-fluorescein conjugates of modified and non-modified crRNA (Table 1 and Table S1, Figure 2). When designing the modified guide RNAs, we took into account the published data on the influence of such modifications on the activity of the CRISPR/Cas9 genomic editing system [9,14,15,16,17,18,19]. We also relied on data about the interactions of 2’-OH groups of guide RNAs with amino acid residues of Cas9 nuclease [17,20,21].

Using the solid-phase phosphoramidite method, we synthesized non-modified guide RNAs (Table 1), a series of modified guide RNAs (Figure 2, Table S1) with pyrimidine ribonucleotides replaced with 2’-fluoro analogs (F1, trF1, sgF1) and a series of modified guide RNAs with deoxyribonucleotides in selected positions (D1, D2, D3_1, D3_2, D3_3, trD1, sgD1).

Two guide RNAs, D1 and D2, differed from series D3 by the additional substitution of ribonucleotides with deoxyribonucleotides from the 22nd to the 27th nucleotide region. The D3_1, D3_2 and D3_3 crRNAs have different quantities of deoxyribonucleotides at the 5’-end. In tracrRNA trD1, more than half of the ribonucleotides were replaced with deoxyribonucleotides. In a series of 2’-O-methylated guide RNAs, selected ribonucleotides were replaced with 2’-O-methylated ones. Guide crRNA M2 contained an «inverted» thymidine (iT) at the 3’-end, attached via 3’-3’-linkage (Figure S1). Furthermore, we constructed crRNA L1 with uridine residues replaced with LNA thymidines (LNA—locked nucleic acid, T^LNA^).

We also synthesized 3’-fluorescein derivatives of modified and non-modified crRNAs (Table S1, Figure S2). In the case of crRNA M2 containing iT at the 3’-end, the fluorescein label was attached to the 5’-end (Figure S2).

### 2.2. Nuclease Resistance of Modified crRNAs

Nuclease resistance is one of the essential characteristics determining the possibility of in vitro and in vivo applications for therapeutic nucleic acids. Model serum-containing solutions are widely used for fast and convenient testing of stability of modified oligonucleotides in biological media [22].

**Figure 3:**
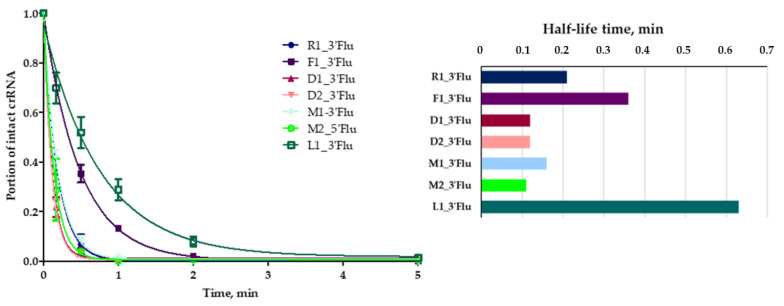
The curves and half-life times of crRNAs degradation in a model system containing 10% fetal bovine serum in IMDM medium. Conditions: crRNA concentration 1 µM, 37 °C, 1 h. All experiments were repeated in triplicate.

We investigated the influence of crRNAs’ chemical modifications on their nuclease stability using fluorescently labeled crRNAs. A comparative study was carried out in a model system containing 10% fetal bovine serum in Iscove’s Modified Dulbecco’s Medium (IMDM) (Figure 3).

We estimated the dynamics of fluorescent crRNA nuclease digestion using gel electrophoresis and fluorescent visualization (Figure S3) and determined the rate constants and half-life times for RNA cleavage (Table 2). The lowest rate constants were found for crRNA F1 with 2’-fluoro nucleotides and crRNA L1 with T^LNA^ monomers. Therefore, we concluded that these particular types of modification significantly increase the stability of guide RNAs.

The rate constants and half-life times were the same for guide crRNA D1_3’Flu and crRNA D2_3’Flu. Therefore, the replacement of four 5′-terminal ribonucleotides with deoxyribonucleotides did not significantly affect serum stability.

We observed a different manner of modified RNA degradation by serum nucleases. RNAs R1, M1, M2 and L1 degraded completely after several minutes of incubation in the model system. On the contrary, crRNAs D1, D2 and F1 rapidly lost only two 5’-terminal nucleotides, and the resulting 5′-shortened molecules remained stable during 1 h of incubation (Figures S3 and S4). We also registered a very fast degradation of crRNA M2 with 3′-terminal iT, but this effect was probably due to the quick removal of the 5’-fluorescein residue from crRNA. This hypothesis was proven by the visualization of crRNA after nuclease digestion with «Stains-all» dye staining. It was shown that crRNA M1 was stable for the first minute, and crRNA M2—for up to five minutes of incubation in the presence of serum nucleases.

The preliminarily assembled gRNA complex with Cas9 protein ordinarily used for in vitro experiments and for in vivo targeted delivery employs different types of carriers (liposome, nanoparticles, nucleoprotein particles) [23,24,25,26], which enhance the stability of guide RNAs and sufficiently increase their half-life time.

Therefore, the obtained results prove that the 2’-fluoro and T^LNA^ modified crRNA are most stable among the investigated variants of modified crRNA. The next obligatory requirement for the successful application of modified guide RNAs in a CRISPR/Cas9 system is their good ability to induce DNA cleavage by the Cas9 nuclease.

### 2.3. Efficacy of the Plasmid Cleavage

We estimated the influence of RNA modifications on the functional activity of a CRISPR/Cas9 system using plasmid DNA as a model dsDNA substrate. The pBS2SKM plasmid at the base of pBluescript II SK(-) contained the protospacer sequence required for introducing the guide crRNA or sgRNA to the dsDNA target and the adjacent fragment (5’-TGG-3’) to the protospacer adjacent motif (PAM). The protospacer sequence originated from prophage-encoded gene speM, a superantigen-encoding gene [10,27].

The CRISPR/Cas9 system employed in our study contains the Cas9 protein and two variants of synthetic guide RNA: (1) a pair of crRNA and tracrRNA or (2) one modified sgRNA. The cleavage was carried out for 1 h at 37 °C with the guide RNA: Cas9: plasmid ratio set at 50:50:1. The products of the DNA cleavage were analyzed in agarose gel with ethidium bromide visualization. The initial DNA plasmid presented mainly as a supercoiled form with a small fraction of relaxed form (Figure S5). The cleavage of supercoiled plasmid DNA resulted in the formation of linear plasmid DNA containing breaks in both chains. No cleavage occurred in the absence of guide RNAs (Figure S6).

**Figure 4:**
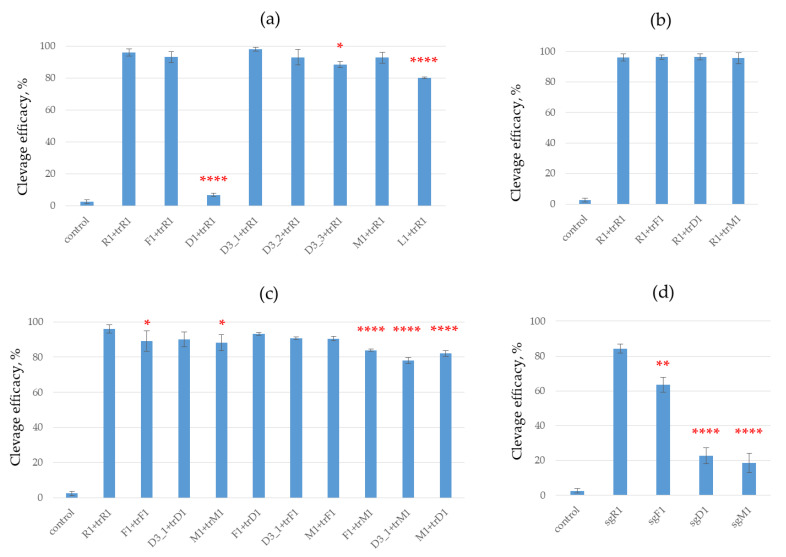
The cleavage efficacy of model plasmid pBS2SKM Psp2 TTG by nuclease Cas9 in the presence of modified guide RNAs. (**a**) Modified crRNAs and non-modified tracrRNA. (**b**) Non-modified crRNA and modified tracrRNAs. (**c**) Combinations of modified crRNAs and tracrRNAs. (**d**) Modified sgRNAs. Control corresponds to the cleavage extent in the absence of guide RNAs and Cas9 nuclease. The conditions are presented in the Materials and Methods section. *p*-values are represented by asterisks, *—*p* < 0.05, **—*p* < 0.01, ****—*p* < 0.0001 compared to positive control R1 + trR1 (a,b,c) or sgR1 (d), by one-way ANOVA with Dunnett’s post hoc test (n = 3). Error bars as s.d.

To compare the effect of crRNA modification, we performed DNA cleavage with Cas9 nuclease in the presence of a dual guide RNA system (crRNA and tracrRNA) containing different variants of modified crRNA and the same non-modified tracrRNA (Figure 4a). Guide crRNA F1 with 2’-fluoro nucleotides, crRNA L1 with LNA nucleotides, crRNA with 2’-O-methylated nucleotides (M1 and M2) and crRNAs D3_1, D3_2, D3_3 with deoxyribonucleotides provided nearly the same efficacy of plasmid DNA cleavage as non-modified crRNA. The introduction of deoxyribonucleotide in the 22–27 region of crRNA led to the total disappearance of DNA cleavage activity, which coincides nicely with the data of Yin and co-authors [18] but disagrees with the work of O’Reilly and co-authors [9]. According to Ref [28], this RNA fragment interacts with Cas9 protein, and its modification is highly undesirable.

The use of crRNA D3_1 modestly improved the DNA cleavage efficacy. The participation of crRNA D3_3 or M1 in the Cas9 complex slightly reduced the cleavage efficacy compared to non-modified crRNA (R1). Meanwhile, we observed a remarkable decrease in the case of crRNA L1 (Figure 4a).

In the D3 series, the cleavage efficacy decreased with the number of deoxyribonucleotides, from 0 (D3_1) to 4 deoxyribonucleotides (D3_2) and 8 deoxyribonucleotides at the 5’-end. This effect probably originates from destabilization of a hybrid duplex between crRNA and the protospacer sequence in the DNA target due to the replacement of RNA nucleotides with DNA ones, in analogy with Refs [29,30].

To determine the influence of the 3’-terminal fluorescein label introduced in guide crRNA on CRISPR/Cas9 system efficacy, we tested R1_3’Flu as a component of the gene editing system. According to the results, fluorescent 3′-labeling does not induce any significant changes in the ability of the CRISPR/Cas9 system to cleave model plasmid DNA (Figure S7).

We then estimated the effect of tracrRNA modification on Cas9 cleaving activity. In these series of experiments, we examined the efficacy of plasmid DNA cleavage by Cas9 using non-modified crRNA combined with different types of modified tracrRNA (Figure 4b). DNA cleavage efficacy in the CRISPR/Cas9 system remained nearly the same in all cases. All three types of modified tracrRNA, trM1, trD1 and trF1, worked equally well in this system.

In the next step, the modified crRNAs F1, D3_1 and M1 were combined with modified trarcRNA trF1, trD1 and trM1 and used as guide RNAs in an in vitro system. In most cases, the use of crRNA/tracrRNA combinations did not significantly change the cleavage efficacy (Figure 3c). A statistically significant decrease was observed for the combinations M1 with trD1 and for D3_1 or F1 with trM1 as compared to positive control R1 + trR1 (Figure 3c). A slight decrease was registered for RNA combinations F1 + trF1 and M1 + trM1 compared to the same control (Figure 4c).

We also investigated the effect of modified sgRNA on the activity of CRISPR/Cas9. Three modified sgRNAs containing 2’-fluoro (sgF1), 2’-O-methyl (sgM1) and deoxyribonucleotides (sgD1) and non-modified sgRNA sgR1 were tested in these experiments. The cleavage efficacy decreased substantially for the CRISPR/Cas9 systems containing modified sgRNAs sgD1 and sgM1 (Figure 4d). The system with sgF1 showed higher cleavage activity (the efficacy was about 60% after 1 h of incubation) than the system with sgM1 or sgD1.

The obtained results allow comparing the influence of different types of guide RNA modifications on the functional activity of the CRISPR/Cas9 system. In most cases, our results are in good correlation with the literature data. For the first time, we compared LNA-modified crRNA with other types of modified crRNA. We confirmed the data of Yin and co-authors [18] concerning the disappearance of the activity of Cas9 protein upon modifications of nucleotides 22–27 from the 5’-end within the crRNA.

We observed a very prominent increase in the activity of CRISPR/Cas9 systems containing modified sgRNA. Moreover, the use of modified crRNA, tracrRNA or their combination is also very promising due to their high cleavage activity.

### 2.4. Kinetics of Plasmid Cleavage by CRISPR/Cas9 System Containing Modified crRNA

**Figure 5:**
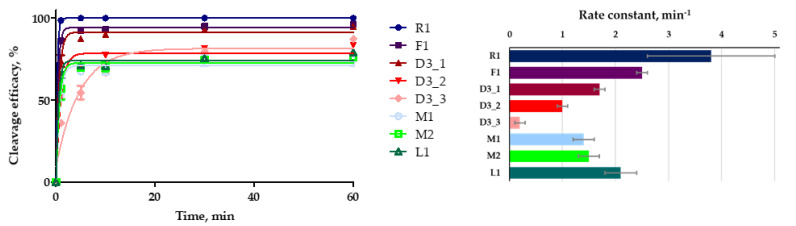
Kinetic curves and rate constants of plasmid cleavage by CRISPR/Cas9 systems containing modified crRNA and their unmodified analog. Plasmid cleavage curves for CRISPR/Cas9 systems containing various modified crRNAs were plotted from at least three independent experiments.

We also compared the kinetics of plasmid DNA cleavage by a CRISPR/Cas9 system driven by modified crRNA. In these studies, we used a lower excess of Cas9 protein and guide RNAs than in the previous experiments described above. Kinetic experiments were performed on the model plasmid DNA with the ratio of Cas9:guide RNAs:plasmid DNA set at 15:15:1. The efficacy of plasmid cleavage was analyzed in the same manner as for the previous experiments. The aliquots were taken from the reaction mixture after certain time points and analyzed by agarose gel electrophoresis (Figure S8). The kinetic curves of plasmid cleavage are presented in Figure 5.

The modified crRNAs F1 and D3_1 were the most effective in this set. All modified crRNAs provided lower rate constants than the native crRNA R1 (Table 3).

The cleavage rate constants for the CRISPR/Cas9 system containing crRNAs F1 and D3_1 were 2.5 min^−1^ and 1.7 min^−1^, respectively. The system with L1 showed a rate constant of 2.1 min^−1^, while the cleavage efficacy was approx. 20% lower as compared to F1, D3_1 or control non-modified R1 crRNA (Table 3).

A downward trend in the efficacy and rate constant of cleavage was also observed for the D3 crRNA series (Table 3). This effect can also be explained by destabilization of a hybrid duplex between crRNA and the protospacer sequence in the DNA target due to the presence of deoxyribonucleotides in the crRNA.

The modifications of crRNA slowed down the DNA cleavage by Cas9 nuclease in the in vitro system, but the pronouncement of this effect depends on the particular type and pattern of modification. For 2’-fluoro, LNA-modified crRNA and one variant of DNA-modified crRNA (D3_1), the decrease in the cleavage rate is not prominent, as distinct from the clearly unfavorable 2’-O-methyl modifications and extensive DNA modifications of crRNA (D3_2 and D3_3).

### 2.5. Influence of crRNA 2’-Modifications on the Specificity of DNA Cleavage

**Figure 6:**
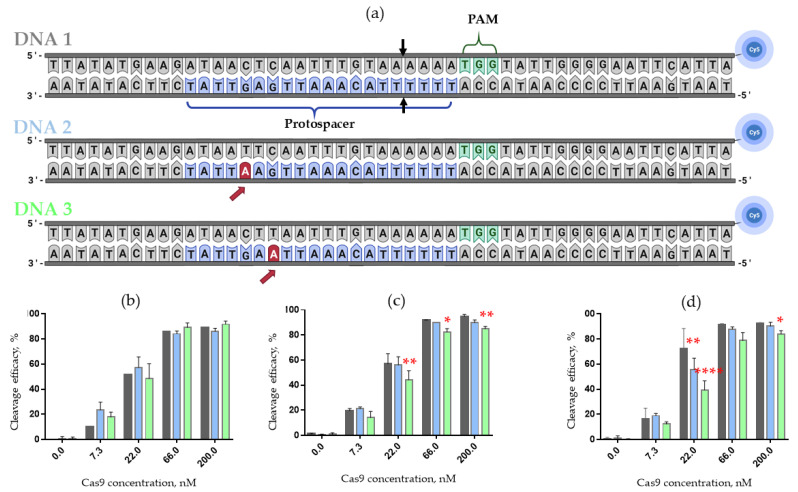
(**a**) The model synthetic DNA duplexes bearing Cy5 fluorescent label at 3′-end of non-target chain, varied by base pairs at positions 5 and 7 of protospacer (DNA1, DNA2, DNA3). PAM—protospacer adjacent motif. Black arrows indicate the cleavage sites in DNA target; red arrows point to the mismatches. The cleavage efficacy of the synthetic DNA duplexes by nuclease Cas9 in the presence of non-modified R1 crRNA (**b**), 2′-F modified F1 crRNA (**c**) and LNA-modified L1 crRNA (**d**). The data are obtained from at least three independent experiments. Gray columns—cleavage efficiency of DNA1; blue columns—cleavage efficiency of DNA2; green columns—cleavage efficiency of DNA3. The conditions are presented in the Materials and Methods section. *p*-values are represented by asterisks, *—*p* < 0.05, **—*p* < 0.01, ****—*p* < 0.0001 compared to efficiency of the DNA duplex cleavage by complex Cas9 with non-modified crRNA by two-way ANOVA with Dunnett’s multiple comparison test (n = 3). Error bars as s.d.

The specificity of CRISPR/Cas9 systems driven by 2’-fluoro- and LNA-modified crRNAs, non-modified tracrRNAs and Cas9 protein was examined on three model 50 bp DNA duplexes (Figure 6a). Control duplex DNA1 contained the 20-nucleotide protospacer sequence, fully complementary to a guide crRNA and PAM sequence at the non-target chain. Two other model dsDNA contained single-nucleotide G- > A mismatches within the protospacer sequence, both located in the non-seeded region (Figure 6a, DNA2 and DNA3, mismatch shown by the red arrow). According to the literature, non-specific (off-target) cleavage usually takes place when the mismatch is located in the non-seeded region, where perfect matching between guide RNA and addressed DNA is not so crucial (see, e.g., Refs [20,31]). To monitor the cleavage, we labeled each dsDNA substrate with a fluorescent cyanine 5 (Cy5) dye attached at the 3’-end of the non-target chain.

To analyze the cleavage products, we employed electrophoretic separation of the products in denaturing polyacrylamide gel followed by fluorescence imaging. For better distinction of differences in the cleavage efficacy of DNA duplexes, we used a serial dilution (3-fold, 9-fold and 27-fold) of the Cas9/guide RNAs complexes.

A comparative study of DNA cleavage by the Cas9 complex with 2’-fluoro-modified or LNA-modified crRNA revealed a decrease in the cleavage efficacy of mismatch-containing DNA2 and DNA3 duplexes as compared to fully matched DNA1 (Figure 6b–d). Notably, in the case of the control CRISPR/Cas9 system driven by unmodified crRNA, we did not observe any statistically significant changes in the cleavage efficacy of matched and mismatched dsDNA substrates.

Finally, we can conclude that Cas9 complexes with 2’-fluoro-modified or LNA-modified crRNAs are sensitive to single nucleotide substitutions in model DNA duplexes, which suggests that the off-target effects can be reduced by using these modified crRNAs in the CRISPR/Cas9 system.

## 3. Materials and Methods

### 3.1. Materials

A controlled pore glass support (CPG) derivatized with 2′-O-TBDMS-G, 2′-O-TBDMS-U, deoxycytosine (dC), deoxythymidine (dT) or inverted deoxythymidine (iT), 5′,N-protected 2′-O-TBDMS-ribo (A, C, G or U), 5′,N-protected 2’-O-methyl-ribo (A, C, G or U), 5′,N-protected 2’-fluoro (U, C), LNA (T) and deoxyribo (dA, dC, dG or dT,) nucleoside phosphoramidites, 3’-(6-fluorescein) CPG were purchased from Glen Research Inc (Sterling, VA, USA). Sodium perchlorate, *N*-methylimidazole and 40% aqueous methylamine solution were purchased from Acros Organics (Waltham, MA, USA); Stains-all dye, dichloroacetic acid, acrylamide, *N,N’*-methylenebisacrylamide, ammonium persulfate, 2,6-lutidine, tris(hydroxymethyl)aminomethane and ethidium bromide were purchased from Fluka (Buchs, Switzerland); *N,N,N’,N’*-ethylenediamine tetraacetic acid, xylene cyanol FF and bromophenol blue were purchased from Serva (Heidelberg, Germany); triethylamine trihydrofluoride, triethylamine, 5-ethylthio-1H-tetrazol and ethoxytrimethylsilane were purchased from Sigma-Aldrich (Burlington, MA, USA). All solvents (THF, ethanol, CH_3_CN, CH_2_Cl_2_ (various vendors)) were dried by distillation or by 3 Å molecular sieves and stored over CaH_2_.

Recombinant Cas9 endonuclease and pBS2SKM Psp2 TTG plasmid based on the pBluescript II SK(–) vector that contained the insert of the protospacer and PAM (5’-TGG-3’) were obtained according to the standard protocol [32].

### 3.2. Equipment

We used deionized water (Millipore Simplicity System; Millipore, Burlington, MA, USA) to prepare all aqueous solutions. To vortex the solutions, we used a Thermomixer Comfort (Eppendorf, Hamburg, Germany). Oligonucleotide solutions were concentrated on a vacuum Concentrator Plus (Eppendorf, USA). Optical density at 260 nm of oligonucleotide solutions was measured on a NanoDrop 1000 spectrophotometer (Thermo Fisher Scientific, Waltham, MA, USA). The oligonucleotides were precipitated and centrifuged on MiniSpin Plus centrifuges (Eppendorf, Germany).

### 3.3. Phosphoramidite Synthesis of Oligonucleotides

Oligonucleotides were synthesized by the phosporamidite method on an automated ASM-800 DNA/RNA synthesizer (Biosset, Novosibirsk, Russia) according to the optimized synthetic protocol. Oligonucleotides were deprotected and removed from the polymer support by treatment with 40% methylamine for 15 min at 65 °C with stirring. 2’-F-RNA and 3’-fluorescein containing oligonucleotides were treated with 30% aqueous ammonia at room temperature for 16 h. In the case of oligoribonucleotides and mixed sequences containing ribonucleotides, the 2’-O-TBDMSi protective groups were removed by a freshly prepared NMP-Et_3_N-Et_3_N · 3HF mixture (1.5:0.75:1, *v/v/v*) at 65 °C with stirring for 1.5 h, followed by ethoxytrimethylsilane treatment and precipitation of oligoribonucleotides with diethyl ether.

### 3.4. Purification of Guide RNA and 3’-Fluorescein Conjugates

Deprotected crRNA, tracrRNA, sgRNA and 3’-fluorescein conjugates of crRNA were isolated by 15% denaturing polyacrylamide gel electrophoresis (PAGE) in 0.4 mm gel, followed by elution from the gel with 0.3 M NaOAc solution (pH 5.2) and precipitated by ethanol as sodium salts. Oligonucleotides in the gel were visualized in the light of a UV lamp (λ = 254 nm), when the gel was applied on a DC-Alufolien Kieselgel 60 F254 plate (Merck, Darmstadt, Germany). To determine the molar concentrations of oligonucleotides and their conjugates, we used the corresponding molar extinction coefficients at 260 nm calculated by the IDT OligoAnalyzer™ Tool.

### 3.5. Nuclease Resistance of crRNAs

3’-Fluorescein-labeled crRNAs 10 µM solutions were used for investigation of their resistance to nuclease digestion. crRNAs were incubated in 10% fetal bovine serum (Sigma, USA) in IMDM culture medium (Sigma, Burlington, MA, USA) for 1 h at 37 °C. After fixed times, 1 μL (10 pmol) aliquots of this mixture were taken and added to the stop buffer (8 M urea, 50 мM Na_2_EDTA, 89 мM Tris-borate (pH 8.3), 0.1% SDS) at 0 °C. The reaction products were analyzed by gel electrophoresis in 15% polyacrylamide gel and visualized at 312 nm using the E-Box-CX5 gel documentation system (Vilber Lourmat, Collégien, France). The images were quantified using the Quantity One program (Bio-Rad, Hercules, CA, USA). The cleavage efficacy was calculated by the following equation:

$$P_{t}=\left(P_{0}-P_{\infty }\right)\cdot e^{\left(-K\cdot t\right)}+P_{\infty }$$

where $P_{t}$*—*fraction of non-cleaved crRNA at a certain moment of time, *t*—time in minutes, *K*—crRNA cleavage rate constant, $P_{0}$—fraction of non-cleaved crRNA at the initial moment, $P_{\infty }$—fraction of non-cleaved crRNA in unlimited time. The kinetic parameters of cleavage were determined from the cleavage curves using the GraphPad Prism 7.00 software.

### 3.6. Cleavage of Plasmid DNA by the Cas9 Protein in the Presence of Modified Guide RNAs

The reactions were carried out in a cleavage buffer (10 μL) containing 20 mM HEPES (4-(2-hydroxyethyl)-1-piperazineethanesulfonic acid) (pH 7.5), 100 mM KCl, 1 mM dithiothreitol, 0.5 mM Na_2_EDTA, 2 mM MgCl_2_ and 5% glycerol. The control solution contained all components, except the RNAs and the Cas9 protein. We prepared the solutions containing either a pair of guide RNAs, crRNA (1 μM, 1.35 μL, 1.35 pM) and tracrRNA (1 μM, 1.35 μL, 1.35 pM), or sgRNA (1 μM, 1.35 μL, 1.35 pM) and the Cas9 protein (2.24 μM, 0.602 μL, 1.35 pM) in the cleavage buffer. The mixtures were incubated for 15 min at 37 °C. The plasmid pBS2SKM containing the protospacer and PAM sequence (5’-TGG-3’) (1 μL, 50 ng/mL, 27 fmol) was added to each tube. The reaction mixtures were incubated for 1 h at 37 °C. The reaction was stopped by addition of the Quenching Buffer (2.5 μL) containing 250 mM Na_2_EDTA, 1.2% SDS, 0.01% bromophenol blue and 30% glycerol.

### 3.7. Efficacy of the Plasmid Cleavage

The cleavage of the plasmid by the Cas9 protein in the presence of guide RNAs was analyzed by gel electrophoresis in 1% agarose gel in a TAE buffer (4 mM Tris, 3 mM CH_3_COOH, 0.07 mM Na_2_EDTA). The reaction mixture (10 μL) in the Quenching Buffer (250 mM Na_2_EDTA, 1.2% SDS, 0.01% bromophenol blue and 30% glycerol) was applied to the gel. The DNA marker 1 kb (250 to 10,000 bp dsDNA ladder; SibEnzyme, Nowosibirsk, Russia) was used to compare the mobility of the cleavage products. The gel was stained with an ethidium bromide solution. The initial plasmid and the products of cleavage were visualized using the E-Box-CX5 gel documentation system (Vilber Lourmat, Marne-la-Valee, France). The images were quantified in the Quantity One program (Bio-Rad, Hercules, CA, USA). The portion of the plasmid cleavage was calculated by the following equation:

$$N_{\Sigma }=\frac{I_{lin}+I_{rel}}{I_{lin}+I_{rel}+I_{superc}/k}\;\cdot 100\%,$$

where $N_{\Sigma }$ is the total plasmid cleavage; $I_{lin}$ is the intensity of the band corresponding to the linear form of the plasmid; $I_{rel}$ is the intensity of the band corresponding to the relaxed form of the plasmid; $I_{superc}$ is the intensity of the band corresponding to the supercoiled form of the plasmid; k = 1.14 is the coefficient of staining efficiency of the supercoiled form of DNA relative to the relaxed form [33]. The efficacy of cleavage was determined using the Microsoft Excel software.

### 3.8. Kinetics of Plasmid DNA Cleavage by Cas9 Protein in the In Vitro System

The reactions were carried out in a cleavage buffer (85 μL) containing 20 mM HEPES (4-(2-hydroxyethyl)-1-piperazineethanesulfonic acid) (pH 7.5), 100 mM KCl, 2 mM MgCl_2_, 1 mM dithiothreitol, 0.5 mM Na_2_EDTA and 5% glycerol. The control solution contained all components, except the RNAs and the Cas9 protein. The solutions containing either a pair of guide RNAs, crRNA (1 μM, 3.5 μL, 3.5 pM) and tracrRNA (1 μM, 3.5 μL, 3.5 pM), or sgRNA (1 μM, 3.5 μL, 3.5 pM) and the Cas9 protein (5.8 μM, 1.56 μL, 3.5 pM) in the cleavage buffer were prepared and incubated for 15 min at 37 °C. The plasmid pBS2SKM (8.5 μL, 50 ng/mL, 233 fmol) was added to each tube. The reaction was carried out for 1.5 h at 37 °C. The aliquots were taken after 2, 5, 10, 20, 30, 45, 60 and 90 min and added to the Quenching Buffer.

Aliquots (10 μL) in the Quenching buffer (250 mM Na_2_EDTA, 1.2% SDS, 0.01% bromophenol blue and 30% glycerol, pH 8.0) were loaded on the 1% agarose gel. After staining of the gel by ethidium bromide, the products were visualized using the E-Box-CX5 gel documentation system (Vilber Lourmat, Marne-la-Valee, France). The images were quantified using the Quantity One program (Bio-Rad, Hercules, CA, USA). The portion of the plasmid cleavage was calculated as described above. The kinetic parameters of plasmid cleavage efficacy were calculated by the following equation:

$$P_{t}=\left(P_{0}-P_{\infty }\right)\cdot e^{\left(-K\cdot t\right)}+P_{\infty }$$

where $P_{t}$*—*fraction of cleaved DNA plasmid at the moment, *t*—time in minutes, *K*—DNA plasmid cleavage rate constant, $P_{0}$—fraction of cleaved DNA plasmid at the initial moment, $P_{\infty }$—fraction of cleaved DNA plasmid in unlimited time. The kinetic parameters of cleavage were determined from the cleavage curves using the GraphPad Prism 7.00 software.

### 3.9. Cleavage of Model Fluorescently Labeled DNA Duplexes by the Cas9 Protein in the Presence of Modified crRNAs

The DNA duplex cleavage reaction was performed in 10 μL of reaction buffer (20 μM HEPES, (pH 7.5), 100 mM KCl, 1 mM DDT, 10 mM MgCl_2_, 5% glycerol, 0.2 mg/mL polyA). The complex of guide RNAs (crRNA and tracrRNA) was formed by combining 7.5 μL crRNA (1.76 μM, 13.2 pmol) and 7.5 μL tracrRNA (1.76 μM, 13.2 pmol). The complex was incubated for 5 min at 90 °C, cooled to room temperature and then diluted to a concentration of 440 nM. The final concentration of the (crRNA + tracrRNA):Cas9 complex was 220 nM. The RNAs/Cas9 were than serially diluted 3, 9, 27 times, and 9 μL of the solution was taken from each dilution (220 nM, 2.0 pmol; 73 nM, 0.66 pmol; 24 nM, 0.22 pmol; 8.1 nM, 0.073 pmol) and added to 1 μL (100 nM, 0.1 pmol) of Cy5-labeled DNA duplex water solution. The reaction mixture was incubated for 1 h at 37 °C, and then, the reaction was quenched by adding 20 μL of the stop buffer (3.33 mM Na_2_EDTA, 0.017% SDS, 3.33% glycerol in formamide).

### 3.10. Efficacy of Model DNA Duplex Cleavage

The cleavage of model DNA duplexes by the Cas9 protein in the presence of guide RNAs was analyzed by gel electrophoresis in 15% denaturing polyacrylamide gel and visualized by fluorescence of Cy5 on 600 nm using the Amersham Typhoon fluorescent scanner (Cytiva, Uppsala, Sweden). The images were quantified using the Quantity One program (Bio-Rad, USA). The percent of DNA duplex cleavage was calculated by the following equation:

$$N=\frac{I_{product}}{I_{product}+I_{duplex}}\;\cdot 100\%$$

where N is the DNA cleavage; $I_{product}$ is the intensity of the band corresponding to the cleavage product; $I_{duplex}$ is the intensity of the band corresponding to the initial dsDNA. The obtained values were further processed by Microsoft Excel software.

### 3.11. Statistical Analysis of the DNA Cleavage by CRISPR/Cas9 System

The outcome variables are expressed as mean standard deviations (SDs). Each experiment was repeated at least three times. Statistical analysis was performed using GraphPad Prism 7.00 (GraphPad Software, San Diego, CA, USA). We used the one-way analysis of variance (one-way ANOVA) test to compare the means of plasmid cleavage efficiency with the different guide RNA variants. Dunnett’s post hoc test was used for comparative analysis of the means of cleavage efficiency with the control mean (with non-modified RNA). A two-way ANOVA test was used to compare the means of DNA duplex cleavage efficiency depending on the type of DNA duplex and Cas9 concentration. Dunnett’s post hoc test was used to compare the means of cleavage efficiency to the control mean (a completely complementary DNA duplex). Differences were considered significant if the *p*-value was <0.05.

## 4. Conclusions

The chemical synthesis of RNA allows for incorporating site-specific chemical modifications in guide RNAs for the CRISPR/Cas9 system. This approach was used for improving DNA editing in terms of efficacy, specificity, in vivo and in vitro stability, cellular delivery and avoiding the immune system [5,34]. The particular type and pattern of chemical modifications can be selected on the basis of data regarding the structure of the guide RNAs/Cas9 protein complex, interactions of guide RNAs with the Cas9 protein [28,35,36] and experimental results on modified guide RNA properties in CRISPR/Cas9 systems [9,14,15,16,17,18,19,34]. The lessons learned from early proposed RNA-based gene expression regulation technologies [37,38,39] can also be taken into account upon the design of modified guide RNAs.

A comparative in vitro study of differently modified synthetic guide RNAs shows that 2’-fluoro and LNA modifications improve nuclease stability and do not sufficiently change the efficacy of the CRISPR/Cas9 system. Our pattern of tracrRNA modifications preserved the cleavage activity of Cas9 protein. Extensive modification of sgRNA by 2’-O-methylribonucleotides or 2’-deoxyribonucleotides prominently decreased the efficacy of DNA cleavage. The 2’-fluorinated sgRNA still possessed good activity in the CRISPR/Cas9 system. Introducing deoxyribonucleotides into RNA positions responsible for interaction with Cas9 protein reduced the efficacy of DNA cleavage. The crRNA modifications in the region that forms a duplex with the DNA target are crucial for the total efficacy and rate constant of a CRISPR/Cas9 system. Selective modification within this region of crRNA or sgRNA can also improve the specificity of CRISPR/Cas9 action and reduce the off-target effects.

2′-modified oligonucleotides provide a set of favorable properties to synthetic nucleic acids, such as high metabolic stability, better affinity to target NA and a better toxicity profile [40,41,42,43]. They are now generally considered as potential next-generation therapeutics.

Undoubtedly, additional investigations are necessary to reveal the best variants of chemically modified guide RNAs for their future practical applications. Improving the specificity of CRISPR/Cas9 by the chemical modifications of guide RNAs to avoid off-target effects also represents an object of intense investigations.

## Figures and Tables

**Table 1:** Sequences of guide RNAs.

Name	Sequence, 5’-3’
R1	AUAACUCAAUUUGUAAAAAAGUUUUAGAGCUAUGCUGUUUUG
trR1	AACAGCAUAGCAAGUUAAAAUAAGGCUAGUCCGUUAUCAACUUGAAAAAGUGGCACCGAGUCGGUGCUUUUUUU
sgR1	GGAUAACUCAAUUUGUAAAAAAGUUUUAGAGCUAGAAAUAGCAGUUAAAAUAAGGCUAGUCCGUUAUCAACUUGAA-AAAGUGGCACCGAGUCGGUGCUUUU

The fragment corresponding to the complementary interaction with DNA target is underlined.

**Table 2:** Guide crRNA half-life times and rate constants of their cleavage in the presence of serum nucleases.

	D2_3’Flu	D1_3’Flu	R1_3’Flu	F1_3’Flu	M1_3’Flu	M2_5’Flu	L1_3’Flu
K, min^−1^	5.7 ± 1.4	5.6 ± 1.4	3.2 ± 0.6	1.9 ± 0.19	4.5 ± 0.6	6.1 ± 3.4	1.1 ± 0.13
half-life time, min	0.12	0.12	0.21	0.36	0.16	0.11	0.63

**Table 3:** Kinetic parameters of plasmid DNA cleavage by CRISPR/Cas9 system with modified crRNA.

					crRNA			
	R1	D3_1	D3_2	D3_3	M1	M2	L1	F1
P, %	97.5 ± 1.2	91.4 ± 1.1	78.6 ± 1.6	81.5 ± 4.4	71.3 ± 1.5	72.8 ± 1.5	74.1 ± 1.0	94.2 ± 0.5
K, min^−1^	3.8 ± 1.2	1.7 ± 0.1	1.0 ± 0.1	0.2 ± 0.1	1.4 ± 0.2	1.5 ± 0.2	2.1 ± 0.3	2.5 ± 0.1

P—efficacy of plasmid DNA cleavage.

## Supplementary Materials

The following are available online at https://www.mdpi.com/article/10.3390/ijms232113460/s1.

## References

[1] Guzmán N.M., Esquerra-Ruvira B., Mojica F.J.M. (2021). Digging into the lesser-known aspects of CRISPR biology. Int. Microbiol..

[2] Bharathkumar N., Sunil A., Meera P., Aksah S., Kannan M., Saravanan K.M., Anand T. (2021). CRISPR/Cas-Based Modifications for Therapeutic Applications: A Review. Mol. Biotechnol..

[3] Hariprabu K.n.g., Sathya M., Vimalraj S. (2021). CRISPR/Cas9 in cancer therapy: A review with a special focus on tumor angiogenesis. Int. J. Biol. Macromol..

[4] Javaid N., Choi S. (2021). CRISPR/Cas System and Factors Affecting Its Precision and Efficiency. Front. Cell Dev. Biol..

[5] Filippova J., Matveeva A., Zhuravlev E., Stepanov G. (2019). Guide RNA modification as a way to improve CRISPR/Cas9-based genome-editing systems. Biochimie.

[6] Basila M., Kelley M.L., Smith A.V.B. (2017). Minimal 2’-O-methyl phosphorothioate linkage modification pattern of synthetic guide RNAs for increased stability and efficient CRISPR-Cas9 gene editing avoiding cellular toxicity. PLoS ONE.

[7] Hendel A., Bak R.O., Clark J.T., Kennedy A.B., Ryan D.E., Roy S., Steinfeld I., Lunstad B.D., Kaiser R.J., Wilkens A.B. (2015). Chemically modified guide RNAs enhance CRISPR-Cas genome editing in human primary cells. Nat. Biotechnol..

[8] Rahdar M., McMahon M.A., Prakash T.P., Swayze E.E., Bennett C.F., Cleveland D.W. (2015). Synthetic CRISPR RNA-Cas9–guided genome editing in human cells. Proc. Natl. Acad. Sci. USA.

[9] O’Reilly D., Kartje Z.J., Ageely E.A., Malek-Adamian E., Habibian M., Schofield A., Barkau C.L., Rohilla K.J., DeRossett L.B., Weigle A.T. (2018). Extensive CRISPR RNA modification reveals chemical compatibility and structure-activity relationships for Cas9 biochemical activity. Nucleic Acids Res..

[10] Jinek M., Chylinski K., Fonfara I., Hauer M., Doudna J.A., Charpentier E. (2012). A Programmable Dual-RNA-Guided DNA Endonuclease in Adaptive Bacterial Immunity. Science.

[11] Allen D., Rosenberg M., Hendel A. (2021). Using Synthetically Engineered Guide RNAs to Enhance CRISPR Genome Editing Systems in Mammalian Cells. Front. Genome Ed..

[12] Sun B., Chen H., Gao X. (2021). Versatile modification of the CRISPR/Cas9 ribonucleoprotein system to facilitate in vivo application. J. Control. Release.

[13] Chen Q., Zhang Y., Yin H. (2021). Recent advances in chemical modifications of guide RNA, mRNA and donor template for CRISPR-mediated genome editing. Adv. Drug Deliv. Rev..

[14] Yin H., Song C.-Q., Suresh S., Wu Q., Walsh S., Rhym L.H., Mintzer E., Bolukbasi M.F., Zhu L.J., Kauffman K. (2017). Structure-guided chemical modification of guide RNA enables potent non-viral in vivo genome editing. Nat. Biotechnol..

[15] Mir A., Alterman J.F., Hassler M.R., Debacker A.J., Hudgens E., Echeverria D., Brodsky M.H., Khvorova A., Watts J.K., Sontheimer E.J. (2018). Heavily and fully modified RNAs guide efficient SpyCas9-mediated genome editing. Nat. Commun..

[16] Cromwell C.R., Sung K., Park J., Krysler A.R., Jovel J., Kim S.K., Hubbard B.P. (2018). Incorporation of bridged nucleic acids into CRISPR RNAs improves Cas9 endonuclease specificity. Nat. Commun..

[17] Kartje Z.J., Barkau C.L., Rohilla K.J., Ageely E.A., Gagnon K.T. (2018). Chimeric Guides Probe and Enhance Cas9 Biochemical Activity. Biochemistry.

[18] Yin H., Song C.-Q., Suresh S., Kwan S.-Y., Wu Q., Walsh S., Ding J., Bogorad R.L., Zhu L.J., Wolfe S.A. (2018). Partial DNA-guided Cas9 enables genome editing with reduced off-target activity. Nat. Chem. Biol..

[19] Rueda F.O., Bista M., Newton M.D., Goeppert A.U., Cuomo M.E., Gordon E., Kröner F., Read J.A., Wrigley J.D., Rueda D. (2017). Mapping the sugar dependency for rational generation of a DNA-RNA hybrid-guided Cas9 endonuclease. Nat. Commun..

[20] Jiang F., Doudna J.A. (2017). CRISPR–Cas9 Structures and Mechanisms. Annu. Rev. Biophys..

[21] Doudna J.A., Sternberg S.H., Jinek M., Jiang F., Emine K., Taylor D. (2015). Cas9 Crystals and Methods of Use Thereof. WIPO Patent.

[22] Novopashina D.S., Nazarov A.S., Vorobjeva M.A., Kuprushkin M.S., Davydova A.S., Lomzov A.A., Pyshnyi D.V., Altman S., Venyaminova A.G. (2018). Modified Oligonucleotides for Guiding RNA Cleavage Using Bacterial RNase P. Mol. Biol..

[23] van Hees M., Slott S., Hansen A.H., Kim H.S., Ji H.P., Astakhova K. (2022). New approaches to moderate CRISPR-Cas9 activity: Addressing issues of cellular uptake and endosomal escape. Mol. Ther..

[24] Alghuthaymi M.A., Ahmad A., Khan Z., Khan S.H., Ahmed F.K., Faiz S., Nepovimova E., Kuča K., Abd-Elsalam K.A. (2021). Exosome/Liposome-like Nanoparticles: New Carriers for CRISPR Genome Editing in Plants. Int. J. Mol. Sci..

[25] Alimi L.O., Alyami M.Z., Chand S., Baslyman W., Khashab N.M. (2021). Coordination-based self-assembled capsules (SACs) for protein, CRISPR–Cas9, DNA and RNA delivery. Chem. Sci..

[26] Yan J., Kang D.D., Dong Y. (2021). Harnessing lipid nanoparticles for efficient CRISPR delivery. Biomater. Sci..

[27] Deltcheva E., Chylinski K., Sharma C.M., Gonzales K., Chao Y., Pirzada Z.A., Eckert M.R., Vogel J., Charpentier E. (2011). CRISPR RNA maturation by trans-encoded small RNA and host factor RNase III. Nature.

[28] Nishimasu H., Ran F.A., Hsu P.D., Konermann S., Shehata S.I., Dohmae N., Ishitani R., Zhang F., Nureki O. (2014). Crystal Structure of Cas9 in Complex with Guide RNA and Target DNA. Cell.

[29] Nakano S. (1999). Nucleic acid duplex stability: Influence of base composition on cation effects. Nucleic Acids Res..

[30] Kankia B.I., Marky L.A. (1999). DNA, RNA, and DNA/RNA Oligomer Duplexes: A Comparative Study of Their Stability, Heat, Hydration, and Mg 2+ Binding Properties. J. Phys. Chem. B.

[31] Zheng T., Hou Y., Zhang P., Zhang Z., Xu Y., Zhang L., Niu L., Yang Y., Liang D., Yi F. (2017). Profiling single-guide RNA specificity reveals a mismatch sensitive core sequence. Sci. Rep..

[32] Anders C., Jinek M., Doudna J.A., Sontheimer E.J. (2014). In Vitro Enzymology of Cas9. Methods Enzymol.

[33] Shubsda M.F., Goodisman J., Dabrowiak J.C. (1997). Quantitation of ethidium-stained closed circular DNA in agarose gels. J. Biochem. Biophys. Methods.

[34] Kelley M.L., Strezoska Ž., He K., Vermeulen A., Smith A.V.B. (2016). Versatility of chemically synthesized guide RNAs for CRISPR-Cas9 genome editing. J. Biotechnol..

[35] Jinek M., Jiang F., Taylor D.W., Sternberg S.H., Kaya E., Ma E., Anders C., Hauer M., Zhou K., Lin S. (2014). Structures of Cas9 Endonucleases Reveal RNA-Mediated Conformational Activation. Science.

[36] Jiang F., Taylor D.W., Chen J.S., Kornfeld J.E., Zhou K., Thompson A.J., Nogales E., Doudna J.A. (2016). Structures of a CRISPR-Cas9 R-loop complex primed for DNA cleavage. Science.

[37] Barrangou R., Birmingham A., Wiemann S., Beijersbergen R.L., Hornung V., Smith A.V.B. (2015). Advances in CRISPR-Cas9 genome engineering: Lessons learned from RNA interference. Nucleic Acids Res..

[38] Zhao Y., Shu R., Liu J. (2021). The development and improvement of ribonucleic acid therapy strategies. Mol. Ther.—Nucleic Acids.

[39] Gao M., Zhang Q., Feng X.-H., Liu J. (2021). Synthetic modified messenger RNA for therapeutic applications. Acta Biomater..

[40] Prakash T., Bhat B. (2007). 2-Modified Oligonucleotides for Antisense Therapeutics. Curr. Top. Med. Chem..

[41] Prakash T.P. (2011). An Overview of Sugar-Modified Oligonucleotides for Antisense Therapeutics. Chem. Biodivers..

[42] Janas M.M., Jiang Y., Schlegel M.K., Waldron S., Kuchimanchi S., Barros S.A. (2017). Impact of Oligonucleotide Structure, Chemistry, and Delivery Method on In Vitro Cytotoxicity. Nucleic Acid Ther..

[43] Esposito C.L., Van Roosbroeck K., Santamaria G., Rotoli D., Sandomenico A., Wierda W.G., Ferrajoli A., Ruvo M., Calin G.A., de Franciscis V. (2021). Selection of a Nuclease-Resistant RNA Aptamer Targeting CD19. Cancers.

