# Supplementary material for: Improving Stability and Specificity of CRISPR/Cas9 System by Selective Modification of Guide RNAs with 2′-fluoro and Locked Nucleic Acid Nucleotides
Source: Int J Mol Sci. 2022 Nov 3;23(21):13460. doi: 10.3390/ijms232113460 (PMC9655745; doi:10.3390/ijms232113460)
Supplement: Supplementary file 1 [file ijms-23-13460-s001.zip › ijms-1998960-supplementary.pdf]

Supplementary Information

Improving stability and specificity of CRISPR/Cas9 system by selective modification of guide RNAs with 2′-fluoro and locked nucleic acid nucleotides

Lubov Sakovina [1,2], Ivan Vokhtantsev [1,2], Mariya Vorobyeva [1], Pavel Vorobyev [1], and Darya Novopashina [1,*]

[1] Institute of Chemical Biology and Fundamental Medicine SB RAS, Novosibirsk, Russia
[2] Novosibirsk State University, Novosibirsk, Russia
* Correspondence: danov@niboch.nsc.ru

Table S1. Sequences of modified guides RNAs, their fluorescently labeled derivatives, and unmodified analogs.

Name	Sequences, 5'-3'	Yield, %
R1	AUAACUCAAUUUGUAAAAAAGUUUUAGAGCUAUGCUGUUUUG	9
R1_3'Flu	AUAACUCAAUUUGUAAAAAAGUUUUAGAGCUAUGCUGUUUUG-Flu-3'	1
F1	AU^{F}AACUCAAU^{F}U^{F}UGU^{F}AAAAAGUUUUAGAGC^{F}U^{F}AU^{F}GC^{F}U^{F}GU^{F}U^{F}U^{F}U^{F}G	10
F1_3'Flu	AU^{F}AACUCAAU^{F}U^{F}UGU^{F}AAAAAGUUUUAGGC^{F}U^{F}AU^{F}GC^{F}U^{F}GU^{F}U^{F}U^{F}U^{F}G-Flu-3'	6
D1	AUAACUCAAUUUGUAAAAAAgttttagagctatgctgttttg	13
D1_3'Flu	AUAACUCAAUUUGUAAAAAAgttttagagctatgctgttttg-Flu-3'	10
D2	ataaCUCAAUUUGUAAAAAAgttttagagctatgctgttttg	16
D2_3'Flu	ataaCUCAAUUUGUAAAAAAgttttagagctatgctgttttg-Flu-3'	12
D3_1	AUAACUCAAUUUGUAAAAAAgUUUAGagctatgctgttttg	4
D3_2	ataaCUCAAUUUGUAAAAAAgUUUAGagctatgctgttttg	6
D3_3	ataactcaAUUUGUAAAAAAgUUUAGagctatgctgttttg	7
M1	A^{m}U^{m}A^{m}ACUCA^{m}A^{m}U^{m}U^{m}UG^{m}U^{m}AAA^{m}A^{m}AAG^{m}UUUUAGA^{m}G^{m}C^{m}U^{m}A^{m}U^{m}G^{m}C^{m}U^{m}G^{m}U^{m}U^{m}U^{m}U^{m}G^{m}	3
M1_3'Flu	A^{m}U^{m}A^{m}ACUCA^{m}A^{m}U^{m}U^{m}UG^{m}U^{m}AAA^{m}A^{m}AAG^{m}UUUUAGA^{m}G^{m}C^{m}U^{m}A^{m}U^{m}G^{m}C^{m}U^{m}G^{m}U^{m}U^{m}U^{m}U^{m}G^{m}-Flu-3'	2
M1_5'Flu	5'-Flu- A^{m}U^{m}A^{m}ACUCA^{m}A^{m}U^{m}U^{m}UG^{m}U^{m}AAA^{m}A^{m}AAG^{m}UUUUAG-A^{m}G^{m}C^{m}U^{m}A^{m}U^{m}G^{m}C^{m}U^{m}G^{m}U^{m}U^{m}U^{m}U^{m}G^{m}	2
M2	A^{m}U^{m}A^{m}ACUCA^{m}A^{m}U^{m}U^{m}UG^{m}U^{m}AAA^{m}A^{m}AAG^{m}UUUUAGA^{m}G^{m}C^{m}U^{m}A^{m}U^{m}G^{m}C^{m}U^{m}G^{m}U^{m}U^{m}U^{m}U^{m}G^{m} -iT	2
M2_5'Flu	5'-Flu- A^{m}U^{m}A^{m}ACUCA^{m}A^{m}U^{m}U^{m}UG^{m}U^{m}AAA^{m}A^{m}AAG^{m}UUUUAG-A^{m}G^{m}C^{m}U^{m}A^{m}U^{m}G^{m}C^{m}U^{m}G^{m}U^{m}U^{m}U^{m}U^{m}G^{m}-iT	0.4
L1	AT^{LNA}AACUCAAUT^{LNA}UGT^{LNA}AAAAAAGUUUUAGAGCT^{LNA}AUGCT^{LNA}GUT^{LNA}UUG	17
L1_3'Flu	AT^{LNA}AACUCAAUT^{LNA}UGT^{LNA}AAAAAAGUUUUAGAGCT^{LNA}AUGC-T^{LNA} GUT^{LNA}UUG-Flu-3'	7
trR1	AACAGCAUAGCAAGUUAAAAUAAGGCUAGUCCGUUAUCAACUUGAAAAAGUGGCACCGAGUCGGUGCUUUUUUU	4

trF1	AAC^{F}AGC^{F}AU^{F}AGC^{F}AAGUUAAAAU^{F}AAGGC^{F}U^{F}AGUC^{F}C^{F}GUUA-U^{F}C^{F}AAC^{F}U^{F}U^{F}GAAAAAGU^{F}GGC^{F}AC^{F}C^{F}GAGU^{F}C^{F}GGUGC^{F}U^{F}U^{F}U^{F}U^{F}U^{F}U^{F}U^F	-
trD1	aacagcatagcaaGUUaAaAtAaggctaGUccGUUAtcAActtgaaaaagtgGcaccgagtcggtgctttt	-
trM1	A^{m}A^{m}C^{m}A^{m}G^{m}C^{m}A^{m}U^{m}A^{m}G^{m}C^{m}A^{m}A^{m}GUUA^{m}AA^{m}AU^{m}AA^{m}G^{m}G^{m}C^{m}U^{m}A^{m}GUC^{m}C^{m}GUUAU^{m}C^{m}AAC^{m}U^{m}U^{m}G^{m}A^{m}A^{m}A^{m}A^{m}A^{m}G^{m}U^{m}G^{m}GC^{m}A^{m}C^{m}C^{m}G^{m}A^{m}G^{m}U^{m}C^{m}G^{m}G^{m}U^{m}G^{m}C^{m}U^{m}U^{m}U^{m}U^m	-
sgR1	GGAUAACUCAAUUUGUAAAAAAGUUUUAGAGCUAGAAAUAGCAAGUAAAAUAAGGCUAGUCCGUUAUCAACUUGAAAAAGUGGCACCGAGUCGGUGCUUUU	-
sgF1	AU^{F}AACUCAAU^{F}U^{F}UGU^{F}AAAAAAGUUUUAGAGC^{F}U^{F}AGAAAU^{F}AGC^{F}AAGUUAAAAU^{F}AAGGC^{F}U^{F}AGUC^{F}C^{F}GUUAU^{F}C^{F}AAC^{F}U^{F}U^{F}GAAAAAGU^{F}GGC^{F}AC^{F}C^{F}GAGU^{F}C^{F}GGU^{F}GC^{F}U^{F}U^{F}U^{F}U^F	-
sgD1	AUAACUCAAUUUGUAAAAAAgUUUUAGagcuagaaauagcaaGUUaAaAtAaggctaGUccGUUAtcAActtgaaaaagtgGcaccgagtcggtgctttt	-
sgM1	A^{m}U^{m}A^{m}ACUCA^{m}A^{m}U^{m}U^{m}UG^{m}U^{m}AAA^{m}A^{m}AAG^{m}UUUUAGA^{m}G^{m}C^{m}U^{m}A^{m}G^{m}A^{m}A^{m}A^{m}U^{m}A^{m}G^{m}C^{m}A^{m}A^{m}GUUA^{m}AA^{m}AU^{m}AA^{m}G^{m}G^{m}C^{m}U^{m}A^{m}GUC^{m}C^{m}GUUAU^{m}C^{m}AAC^{m}U^{m}U^{m}G^{m}A^{m}A^{m}A^{m}A^{m}A^{m}G^{m}U^{m}G^{m}GC^{m}A^{m}C^{m}C^{m}G^{m}A^{m}G^{m}U^{m}C^{m}G^{m}G^{m}U^{m}G^{m}C^{m}U^{m}U^{m}U^{m}U^m	-

N- ribonucleotide, n – deoxyribonucleotide, N^F – 2'-fluoromodified pyrimidine nucleotide, N^m – 2'-O-methylated ribonucleotide, iT – «inverted» thymidine, T^{LNA} – LNA-thymidine, Flu – fluorescein residue.

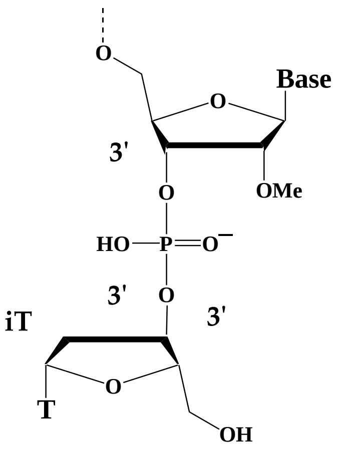

Figure S1. The structure of inverted thymidine attached by 3'-3'-phosphodiester linkage to the crRNA M2.

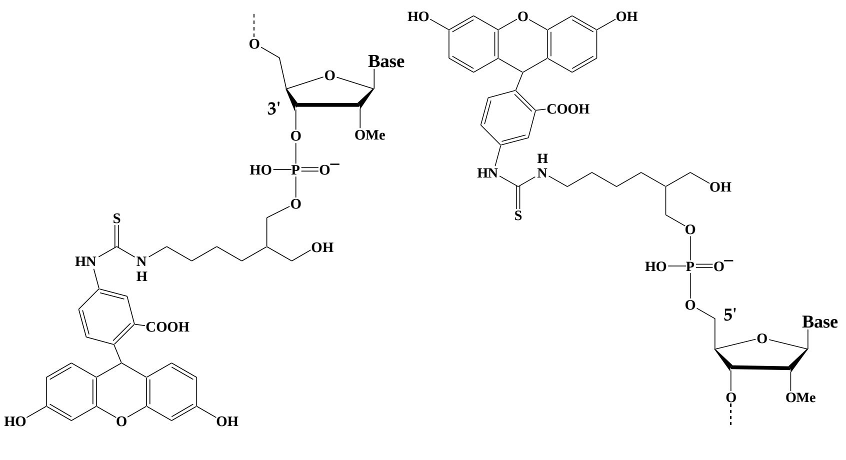

Figure S2. The structures of the conjugates of crRNA with fluorescein residues at 3' or 5'-terminus of crRNAs.

It should be noted that upon cleaving crRNA D1_3'Flu, D2_3'Flu and F1_3'Flu we registered the formation of products several nucleotides shorter than initial crRNA. These fragments remained stable for 1 hour (Figure S3). Probably, it can be explained by the ability of crRNA to form a secondary structure (Figure S4). We suppose that on the first stage of RNA degradation, some ribonucleotides were removed from the 5'-end. Then, the remaining RNA molecule formed a stem that protected 5′-terminal fragment from further degradation. The insertion of deoxyribonucleotides and 2'-fluoromodified nucleotides might significantly stabilize this duplex. As a result, RNA degradation terminates after hydrolysis of two phosphodiester bonds at the 5′-end.

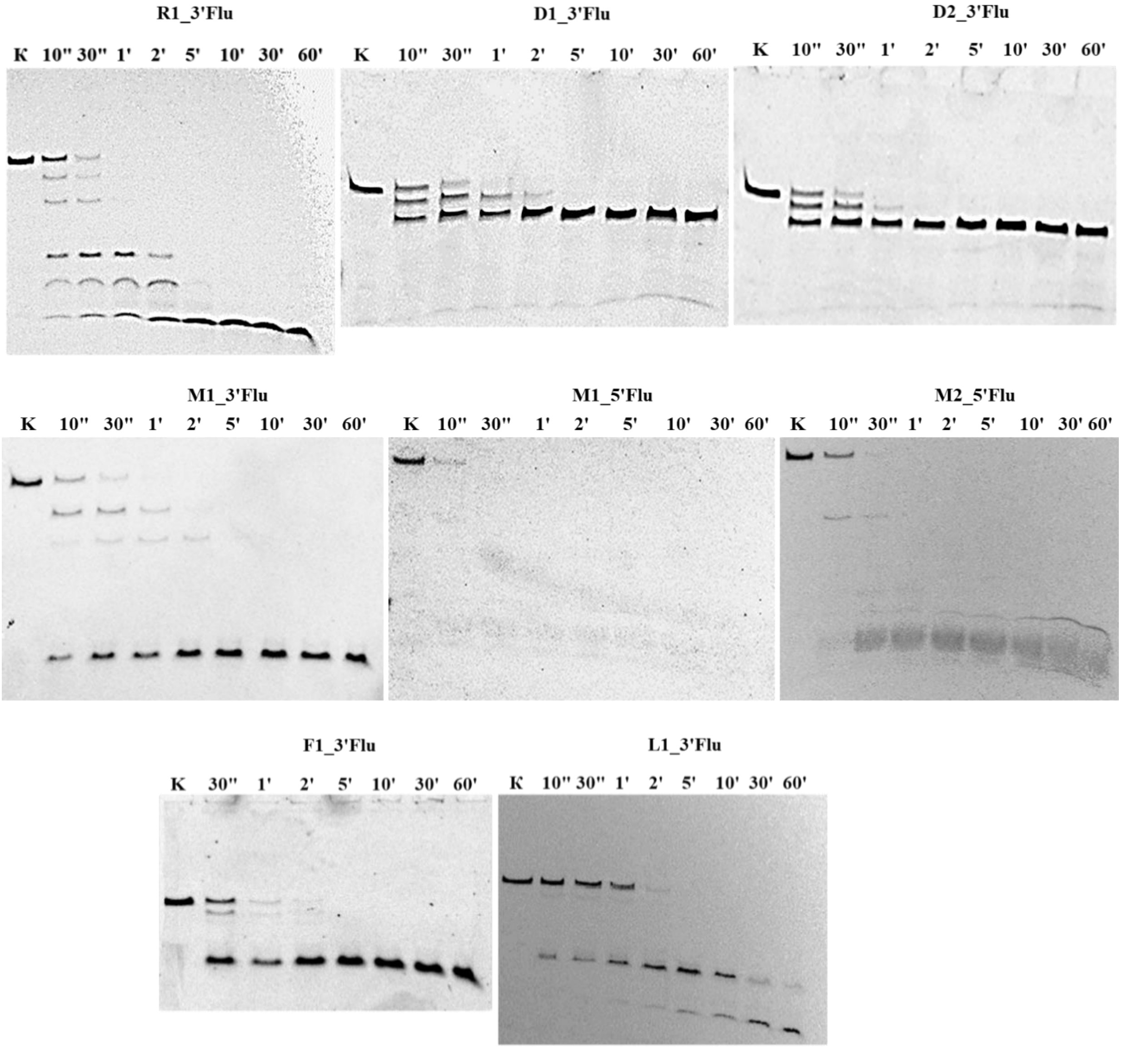

Figure S3. Electropherograms of fluorescently labeled crRNAs cleavage products by serum nucleases in denaturing 15% PAGE. K – control fluorescently labeled crRNA without serum; crRNA was visualized gel documentation system; concentration of fluorescently labeled crRNA in 10% fetal bovine serum was 10^{-6} M; temperature 37°C.

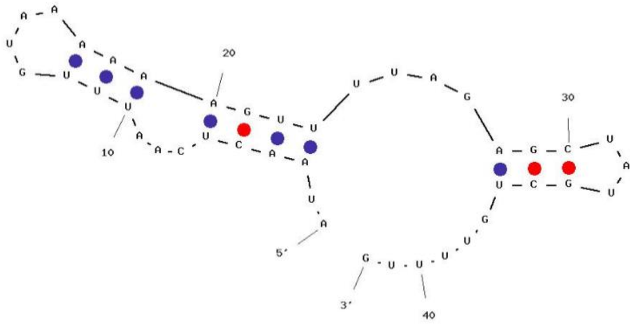

Figure S4. Secondary structure of guide crRNA generated by the OligoAnalyzer Tool service (Integrated DNA Technologies, Inc.).

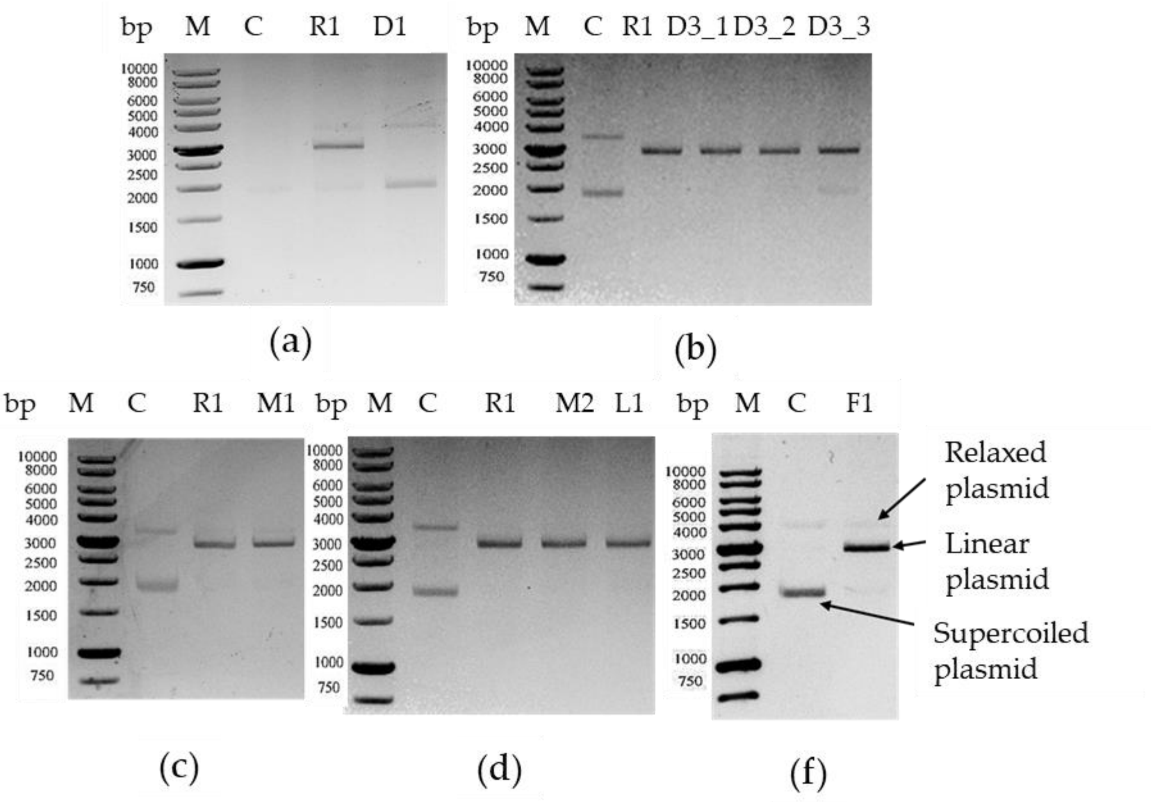

Figure S5. Analysis of plasmid cleavage by CRISPR/Cas9 system in 1% agarose gel. The cleavage efficacy was calculated as the ratio of the linear form of the plasmid to the sum of all its forms in the lane. The calculation was performed using data from at minimum three independent experiments. crRNA: R1, D1 (a), D3_1, D3_2, D3_3 b), M1 (c), M2, L1 (d), F1 (f); C – intact pBS2SKM plasmid; M – DNA marker; visualization by ethidium bromide staining; cleavage conditions 20 mM HEPES (pH 7,5), 100 mM KCl, 1 mM DTT, 0,5 mM Na_2EDTA, 2 mM $MgCl_2$, 5% glycerol, 60 min (T = 37 °C); Cas9 : crRNA + tracrPHK : plasmid DNA 50:50:1.

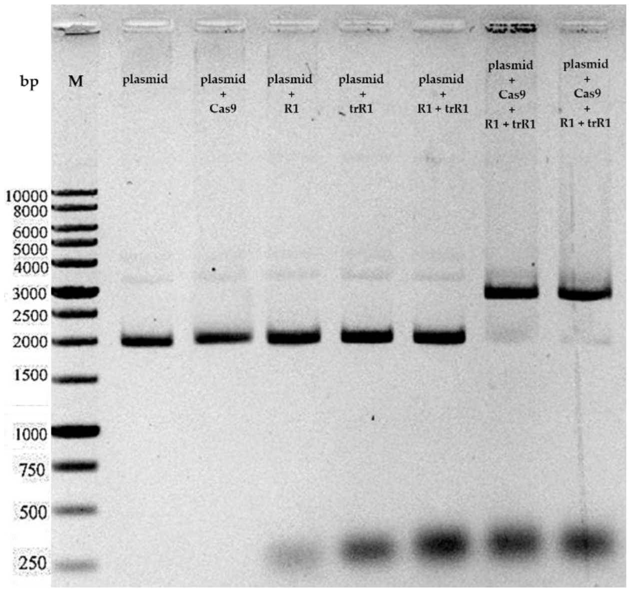

Figure S6. Analysis of plasmid cleavage by CRISPR/Cas9 system in 1% agarose gel. M – DNA marker; plasmid – intact pBS2SKM plasmid in the buffer; visualization by ethidium bromide staining; cleavage conditions 20 mM HEPES (pH 7,5), 100 mM KCl, 1 mM DTT, 0,5 mM Na_2EDTA, 2 mM $MgCl_2$, 5% glycerol, 60 min (T = 37 °C); Cas9 : crRNA + tracrPHK : plasmid DNA 50:50:1.

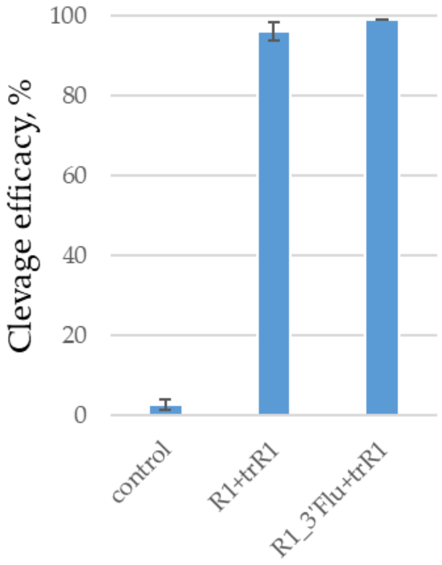

Figure S7. The cleavage efficacy of model plasmid pBS2SKM Psp2 TTG by nuclease Cas9 in the presence of 3'-fluorescein modified guide RNA R1 in comparison with non-modified R1. Control corresponds to the cleavage extent in the absence of guide RNAs and Cas9 nuclease. The conditions are presented in section Materials and Methods. Error bars as s.d.

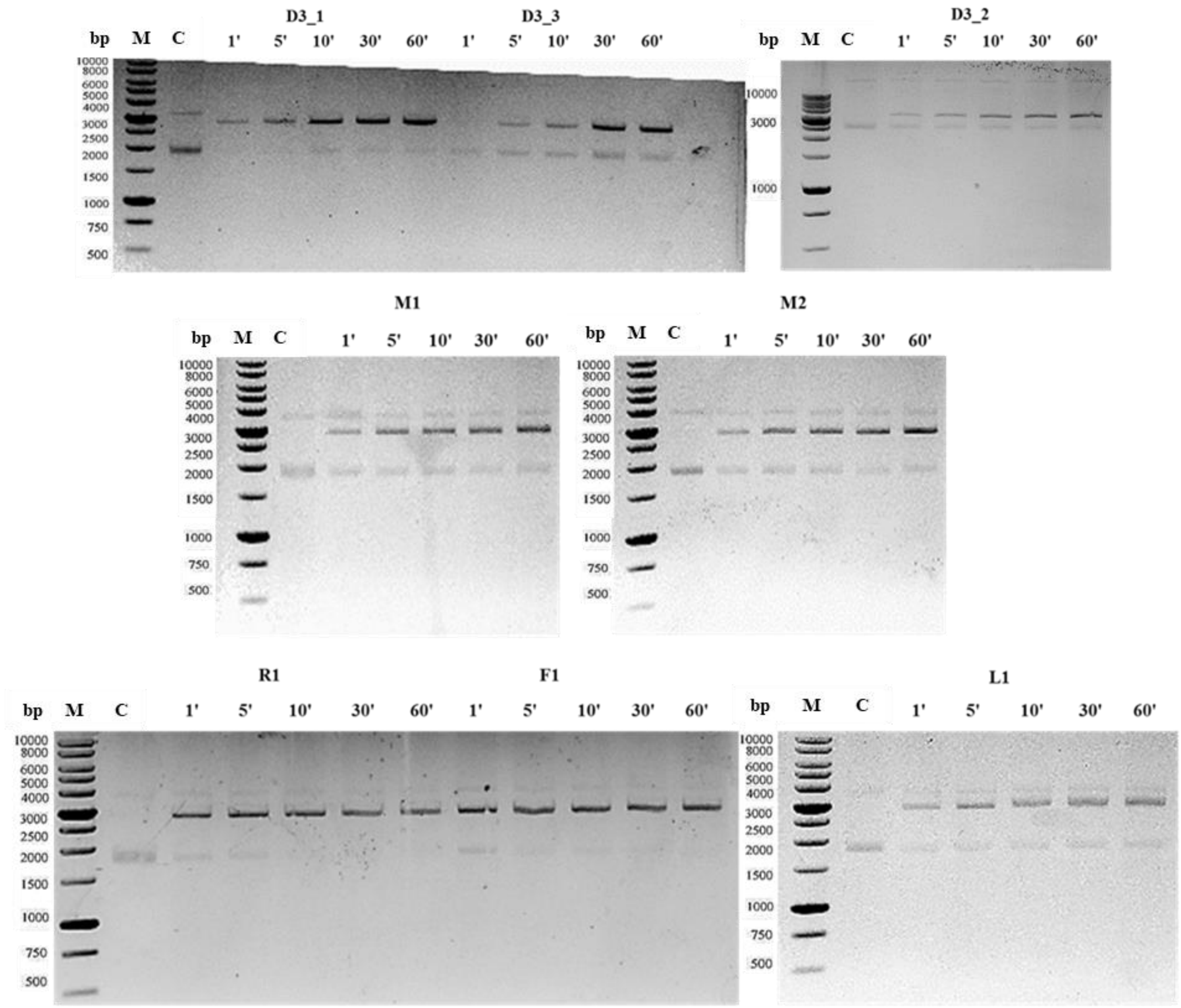

Figure S8. Analysis of cleavage kinetics of pBS2SKM DNA plasmid by CRISPR/Cas9 system with modified crRNAs in 1% agarose gel. The cleavage efficacy was calculated as the ratio of the linear form of the plasmid to the sum of all its forms in the lane. The calculation was performed using data from at minimum three independent experiments. C – untreated pBS2SKM plasmid; M – DNA marker; visualization by ethidium bromide staining; cleavage conditions 20 mM HEPES (pH 7,5), 100 mM KCl, 1 mM DTT, 0,5 mM Na$_2$EDTA, 2 mM MgCl$_2$, 5% glycerol, 60 min (T = 37 °C); Cas9 : crRNA + tracrPHK : plasmid DNA 15:15:1.
